# Anticancer effects of chemokine-directed antigen delivery to a cross-presenting dendritic cell subset with immune checkpoint blockade

**DOI:** 10.1038/s41416-020-0757-2

**Published:** 2020-02-18

**Authors:** Yuki Mizumoto, Hiroaki Hemmi, Masahiro Katsuda, Motoki Miyazawa, Yuji Kitahata, Atsushi Miyamoto, Mikihito Nakamori, Toshiyasu Ojima, Kenji Matsuda, Masaki Nakamura, Keiji Hayata, Yuri Fukuda-Ohta, Masanaka Sugiyama, Tomokazu Ohta, Takashi Orimo, Soichiro Okura, Izumi Sasaki, Koji Tamada, Hiroki Yamaue, Tsuneyasu Kaisho

**Affiliations:** 1 Second Department of Surgery, Wakayama Medical University, Wakayama, Japan; 2 Department of Immunology, Institute of Advanced Medicine, Wakayama Medical University, Wakayama, Japan; 3 Department of Pediatric Oncology, National Cancer Center Hospital, Tokyo, Japan; 4 Department of Immunology, Yamaguchi University Graduate School of Medicine, Ube, Japan

**Keywords:** Immunization, Cancer immunotherapy

## Abstract

**Background:**

Cancer peptide vaccines show only marginal effects against cancers. Immune checkpoint inhibitors (ICIs) show significant curative effects in certain types of cancers, but the response rate is still limited. In this study, we aim to improve cancer peptide vaccination by targeting Ag peptides selectively to a dendritic cell (DC) subset, XCR1-expressing DCs (XCR1^+^ DCs), with high ability to support CD8^+^ T-cell responses.

**Methods:**

We have generated a fusion protein, consisting of an Ag peptide presented with MHC class I, and an XCR1 ligand, XCL1, and examined its effects on antitumour immunity in mice.

**Results:**

The fusion protein was delivered to XCR1^+^ DCs in an XCR1-dependent manner. Immunisation with the fusion protein plus an immune adjuvant, polyinosinic:polycytidylic acids (poly(I:C)), more potently induced Ag-specific CD8^+^ T-cell responses through XCR1 than the Ag peptide plus poly(I:C) or the Ag protein plus poly(I:C). The fusion protein plus poly(I:C) inhibited the tumour growth efficiently in the prophylactic and therapeutic tumour models. Furthermore, the fusion protein plus poly(I:C) showed suppressive effects on tumour growth in synergy with anti-PD-1 Ab.

**Conclusions:**

Cancer Ag targeting to XCR1^+^ DCs should be a promising procedure as a combination anticancer therapy with immune checkpoint blockade.

## BACKGROUND

Over the past few years, cancer immunotherapies have made significant advances.^1^ Immune checkpoint inhibitors (ICIs) showed remarkable clinical effects on various types of cancers by reactivating exhausted T cells.^2–5^ However, ICIs alone have limited anticancer effects, and several combination therapies are being developed. Because ICIs are mainly involved in releasing the brake of anticancer immunity, it can be expected that immune accelerators should show synergistic effects with ICIs. To develop efficient immune accelerators, identification of cancer Ag peptides is important, and various cancer Ag peptides have been so far identified and utilised as anticancer vaccines. Although the clinical effects of such cancer peptide vaccines are limited at present, it should still be useful to generate cost-effective cancer Ag peptide vaccines that can induce effective cytotoxic T-cell (CTL) responses.^6–8^

It can be assumed that Ag peptides are delivered to all types of Ag-presenting cells such as macrophages or dendritic cells (DCs). Such Ag-presenting cells should present Ag peptides to provoke Ag-specific T-cell responses. However, Ag-presenting cells are heterogeneous and divided into several subsets with subset-specific functions, which excel in CD4^+^ or CD8^+^ T-cell responses, or induce regulatory T cells to suppress anticancer immunity.^9^ Classical DC type 1 (cDC1) should be a critical DC subset for Ag peptides to be delivered, because cDC1 is featured by high cross-presentation activity, by which captured Ags can be presented in the context of MHC class I.^10^ cDC1 induces potent anticancer CD8^+^ T cells or CTL responses, and is critical for the anticancer effects of ICIs.^11–14^ In mice, an Ag protein coupling to the monoclonal antibody against an endocytic receptor, DEC205, predominantly expressed on cDC1, leads to Ag delivery to cDC1 and enhancement of CD8^+^ T-cell responses.^15,16^ When combined with anti-CD40 Ab, this Ag delivery induces effective anticancer effects.^17,18^ cDC1 is present beyond species, and defined as CD141^+^ DC in human and CD8α^+^/CD103^+^ DC in mice. Notably, a chemokine receptor, XCR1, is specifically expressed in this DC subset in both, human and mice.^19–22^ Fusion proteins consisting of Ag proteins with an XCR1 ligand, XCL1, were generated and shown to be targeted to cDC1s or XCR1^+^ DCs, and induce efficient CD8^+^ T-cell or CTL responses against viral infection or cancers.^23–25^

In this study, we generated a fusion protein consisting of an Ag peptide instead of an Ag protein, and an XCR1 ligand, XCL1, to target cDC1s to expect selective targeting of Ags to XCR1^+^ DCs. The fusion protein induced potent Ag-specific CD8^+^ T-cell responses in vivo, and showed antitumour effects in both prophylactic and therapeutic models in an XCR1-dependent manner. Notably, pretreatment with the fusion protein could effectively improve the antitumour effects of ICIs. Thus, we have shown that targeting Ag peptides to XCR1^+^ DCs by XCL1 is effective as anticancer vaccines that can be utilised with ICIs. Given the conserved expression pattern of XCR1, peptide delivery targeted to cDC1s or XCR1^+^ DCs should be applicable as a cancer immunotherapy by overcoming the limited effects of ICIs.

## Methods

### Mice

*Xcr1*^*+/venus*^ mice were generated by knocking in the cDNA for a fluorescence protein, Venus, to the *Xcr1* locus, and backcrossed with C57BL/6J mice more than 10 times.^26,27^ *Xcr1*^*venus/venus*^ mice were generated by crossing the *Xcr1*^*+/venus*^ mice, and used as XCR1-deficient mice. C57BL/6J mice and β2-microgloblin (β2m)-deficient mice were purchased from CLEA Japan and Jackson Laboratory, respectively. All mice used were healthy and 7–12-week old, and their body weight was 25 ± 15 g. Under specific pathogen-free conditions, they were housed in plastic cages, with wood chips, which were changed every week, and each cage was kept to five or less heads without mixing gender. The dark/light cycle is 12/12 h, and room temperature is kept at 22 ± 2 °C. All mice were allowed free access to water and sterilised normal chow. The experimental protocols were made in accordance with The Regulations for Animal Experiments in Wakayama Medical University, which states replacement, refinement or reduction (the 3Rs), and approved by Wakayama Medical University Animal Care and Use Committee.

### Reagents

Ovalbumin (OVA) and OVA-derived MHC class I-restricted peptide OVA_253–264_ peptide (SIINFEKL, OT-I peptide) were purchased from Worthington Biochemical and MBL, respectively. Polyinosinic–polycytidylic acid (poly(I:C)) was purchased from InvivoGen. Anti-PD-1 (clone: G4) Ab was previously reported.^28^

### Cell line and cell culture

An OVA-expressing murine B16 melanoma cell line, B16-OVA (clone MO4), was kindly provided by Dr. Senju, Kumamoto University, Kumamoto, Japan.^29^ B16-OVA and human embryonic kidney (HEK) cell line, 293T, were maintained in Dulbecco’s modified Eagle medium (DMEM) supplemented with 10% FBS.

### Generation of a fusion protein, XCL1-OT-I

**Fig. 1:**
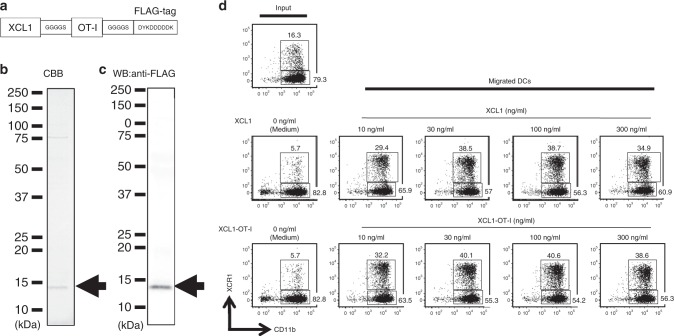
Generation of a fusion protein, XCR1-OT-I. **a** Schematic representation of an amino acid structure of XCL1-OT-I. Murine XCL1 was fused with the OT-I peptide flanked by two glycine-rich linkers, GGGGS. FLAG tag, DYKDDDDK, was attached at the carboxy terminal. **b**, **c** Coomassie Brilliant Blue (CBB) staining (**b**) and western blotting (WB) with anti-FLAG Ab (**c**) of purified XCL1-OT-I. The band for XCL1-OT-I is indicated by arrows. Molecular weight of XCL1-OT-I is estimated as 12.7 kDa. **d** Chemotactic activity of XCL1-OT-I. Flt3L-induced BM DCs were added to the upper chambers of a 24-well Transwell plate, and XCL1 or XCL1-OT-I was added in the lower chambers at the indicated concentrations for 2 h. Cells that migrated into the lower chambers were collected and analysed by a flow cytometer. Numbers indicate percentages of XCR1^+^CD11c^+^ and XCR1^−^CD11c^+^ cells in migrated cells. Similar data were obtained from two independent experiments.

A fusion protein, XCL1-OT-I, was designed as follows (Fig. 1a). First, the cDNA for XCL1-OT-I was generated by ligating murine XCL1 cDNA with cDNA coding the OT-I Ag peptide (SIINFEKL, corresponding to 257th to 264th amino acids of OVA), which was flanked by a glycine-rich linker (GGGGS), and FLAG tag (DYKDDDDK). Then the XCL1-OT-I cDNA was cloned into the pHEK293 Ultra Expression Vector II (TaKaRa), and the expression plasmid for XCL1-OT-I was transfected into HEK293T cells together with the pHEK293 Enhancer Vector (TakaRa) using linear polyethyleneimine (PEI, Sigma-Aldrich). After 16–18 h, the culture medium was changed to serum-free DMEM medium supplemented with 1% Nutridoma (Roche) and 1% sodium pyruvate. XCL1-OT-I protein was purified with anti-FLAG Agarose Affinity Gel (Sigma-Aldrich) from culture supernatants. Purified XCL1-OT-I was subjected to SDS-PAGE electrophoresis with Coomassie Brilliant Blue staining and western blotting with anti-FLAG Ab (Sigma-Aldrich). The molecular weight of XCL1-OT-I protein was estimated as 12.7 kDa.

### Migration assay of XCR1^+^ DCs

To obtain bone marrow (BM)-derived DCs (BM DCs), BM cells were cultured for 8 days in the presence of 100 ng/ml of recombinant human Flt3 ligand (Flt3L, PeptoTech). BM DCs generated by this culture include plasmacytoid DCs (pDCs) and cDCs.^30,31^ cDCs include CD11c^+^CD24^high^CD11b^int^ and CD11c^+^CD24^int^CD11b^high^ cells, which correspond to cDC1s and cDC2s, respectively. XCR1^+^DCs are a major population of cDC1s, but not found in pDCs or cDC2s.^19–22,30,31^ One million BM DCs were placed into the upper chamber of a 24-well Transwell plate (5.0-µm pore size) (Corning). The lower chamber was filled with RPMI1640 with 10% FBS containing murine XCL1 (R&D Systems) or XCL1-OT-I. After 2-h culture, cells in the lower chamber were analysed with FACSVerse (BD Bioscience).

### Detection of OT-I Ag peptide presentation on BM DCs

BM DCs from control (*Xcr1*^*+/venus*^) or XCR1-deficient (*Xcr1*^*venus/venus*^) mice were incubated in the presence or absence of various concentrations of XCL1-OT-I or OVA protein for 6 h, and subjected to FACS analysis. The DCs were gated for CD11c^+^CD24^high^CD11b^int^ and CD11c^+^CD24^int^CD11b^high^ cells, which correspond to cDC1s and cDC2s, respectively.^32^ OT-I Ag peptide presentation was detected with mAb against the OT-I Ag peptide presented on H-2K^b^ (eBio25-D1.16, eBioscience).

### CD8^+^ T-cell responses

Mice were immunised with the indicated doses of OT-I peptides, XCL1-OT-I or OVA protein with or without 20 µg of poly(I:C). At 7 days after immunisation, splenocytes were prepared and subjected to FACS. To detect OT-I Ag-specific CD8^+^ T cells, splenocytes were stained with a H-2K^b^/OVA tetramer (MBL) and mAbs for CD8α, CD49b and CD62L. For intracellular IFN-γ staining, splenocytes were stimulated with 1 µg/ml of OT-I peptides for 6 h in the presence of brefeldin A (10 µg/ml, Sigma-Aldrich), and stained with mAbs for CD8α, CD49b and CD62L. Cells were further fixed with fixation/permeabilisation solution (Cytofix/ Cytoperm Kit, BD Bioscience), and stained with anti-IFN-γ mAb. Percentages of tetramer-positive or IFN-γ^+^ cells among CD8α^+^CD49b^−^CD62L^−^ T cells were calculated.

### FACS analysis

Single-cell suspensions were incubated with CD16/32 mAb (BD Bioscience) to block nonspecific binding of Abs. Dead cells were excluded by staining with a LIVE/DEAD Fixable Dead Cell Stain Kit (Invitrogen). The cells were stained with the following antibodies: APC-Cy7-anti-CD3ε (clone 145-2C11) or PerCP-Cy5.5-anti-CD3ε (clone 145-2C11), PE-Cy7-anti-CD8α (clone 53-6.7), APC-Cy7-anti-CD8α (clone 53-6.7), Alexa Fluor 647-anti-CD8α (clone KT15), APC-Cy7-anti-CD11b (clone M1/70), PE-Cy7-anti-CD11c (clone N418), APC-Cy7-anti-CD11c (clone HL3), PE-anti-CD24 (clone M1/69), PerCP-Cy5.5-anti-B220 (clone RA3-6B2), PE-Cy7-anti-CD62L (clone MEL-14), FITC-anti-CD49b (clone DX5), biotin-anti- or PE-anti-CD103 (clone M290), biotin-anti-H-2Kb–OVA257–264 (clone 25-D1.16), biotin-anti-I-A/I-E (clone M5/114.15.2), PE-anti-CD279 (PD-1, clone J43), APC-anti-IFN-γ (clone XMG1.2) and APC-anti-XCR1 (clone ZET). Biotinylated Abs were visualised by APC or APC-Cy7-conjugated streptavidin. Abs and fluorochrome-conjugated streptavidin were purchased from BD Biosciences, eBioscience, BioLegend and MBL. Stained cells were analysed on a FACSVerse (BD Bioscience) or FACSAria II, and data were analysed with FlowJo software (TreeStar).

### Tumour model

For the prophylactic model, 20 wild-type (C57BL/6J) mice were randomly divided into 4 groups: PBS injection (control), 15 µg (16 µmol) OT-I peptide plus 20 µg of poly(I:C) injection, 6.7 µg (0.16 µmol) OVA protein plus 20 µg of poly(I:C) injection or 2 µg (0.16 µmol) XCL1-OT-I plus 20 µg of poly(I:C) injection on 14 and 7 days before inoculation of B16-OVA cells. Ten β2m-deficient mice were randomly divided into two groups: PBS injection (control) or 2 µg (0.16 µmol) XCL1-OT-I plus 20 µg of poly(I:C) injection on 14 and 7 days before inoculation of B16-OVA cells. Ten XCR1-deficient mice were randomly divided into two groups: PBS injection (control) or 2 µg (0.16 µmol) XCL1-OT-I plus 20 µg of poly(I:C) injection on 14 and 7 days before inoculation of B16-OVA cells.

For the therapeutic model, 20 wild-type (C57BL/6 J) mice were inoculated subcutaneously with B16-OVA cells. Mice were randomly divided into four groups: PBS injection (control), 15 µg (16 µmol) OT-I peptide plus 20 µg of poly(I:C) injection, 6.7 µg (0.16 µmol) OVA protein plus 20 µg of poly(I:C) injection or 2 µg (0.16 µmol) XCL1-OT-I plus 20 µg of poly(I:C) injection subcutaneously on 7 and 14 days after inoculation of B16-OVA cells. Ten β2m-deficient mice were randomly divided into two groups: PBS injection (control) or 2 µg (0.16 µmol) XCL1-OT-I plus 20 µg of poly(I:C) injection subcutaneously on 7 and 14 days after inoculation of B16-OVA cells. Ten XCR1-deficient mice were randomly divided into two groups: PBS injection (control) or 2 µg (0.16 µmol) XCL1-OT-I plus 20 µg of poly(I:C) injection subcutaneously on 7 and 14 days after inoculation of B16-OVA cells.

For analysing the effects of XCL1-OT-I in combination with anti-PD-1 Ab, 20 wild-type (C57BL/6J) mice were inoculated subcutaneously with B16-OVA cells. Mice were randomly divided into four groups: PBS (control) injection, 150 µg of anti-PD-1Ab injection, 2 µg (0.16 µmol) XCL1-OT-I plus 20 µg of poly(I:C) injection or 2 µg (0.16 µmol) XCL1-OT-I plus 20 µg of poly(I:C) plus 150 µg of anti-PD-1Ab injection. PBS or XCL1-OT-I plus poly(I:C) were injected subcutaneously on 14 and 7 days after inoculation of B16-OVA cells. Anti-PD-1 Ab was administered intraperitoneally on days 14, 17 and 21 after inoculation of B16-OVA cells.

Mice were anaesthetised with isoflurane inhalation, and inoculated subcutaneously with B16-OVA cells (5 × 10^5^ cells/100 µl/mouse). Tumour size and mice conditions were monitored every 1, 2 or 3 days. Each group contains five animals, and all the samples were analysed. Tumour size was measured with a calliper, and calculated by the following formula: tumour volume (mm^3^) = (long diameter) × (short diameter)^2^ × 0.5.^33^ There were no significant unexpected adverse events. Mice were humanely killed by cervical dislocation after anaesthesia with isoflurane inhalation when the long diameter of the tumour exceeded 18 mm, or mice showed any signs of too much illness or stress, or at the end of the experiments according to The Regulations for Animal Experiments in Wakayama Medical University.

### Statistical analysis

GraphPad Prism 6 (GraphPad software) was used for statistical analysis. Mann–Whitney U test or ANOVA was used as indicated in the figure legends. Data are shown as mean or mean ± S.E.M. Survival studies were analysed by Kaplan–Meier survival curves and log-rank test. The results were considered statistically significant when the P value was less than 0.05.

## Results

### Generation and characterisation of a fusion protein, XCL1-OT-I

To investigate the potency of direct Ag delivery via chemokine ligands to XCR1^+^ DCs, we designed a fusion protein consisting of murine XCL1 and an Ag peptide. As an Ag peptide, we chose an OVA-derived OT-I peptide, OVA_257–264_ (SIINFEKL), which is presented on MHC class I, H-2K^b^. In the fusion protein, XCL1-OT-I, the OT-I peptide was flanked by glycine-rich linkers and followed by the FLAG tag (Fig. 1a). XCL1-OT-I was produced by HEK-293T cells, purified by anti-FLAG Abs and analysed by Coomassie Brilliant Blue staining (Fig. 1b) and western blots (Fig. 1c). We detected one major band at 12.7 kDa, which is consistent with the predicted size of XCL1-OT-I.

Next, we examined whether XCL1-OT-I has a chemotactic activity, like XCLl does. We then performed a Transwell migration assay by using XCL1-OT-I and XCL1. The results showed that XCR1^+^ DCs dominantly migrated to the lower chamber in response to XCL1-OT-I as well as XCL1. XCL1-OT-I showed an activity to induce selective migration of XCR1^+^ DC in a comparable manner to XCL1 (Fig. 1d). Thus, XCL1-OT-I retained the same chemotactic activity as XCL1 does.

**Fig. 2:**
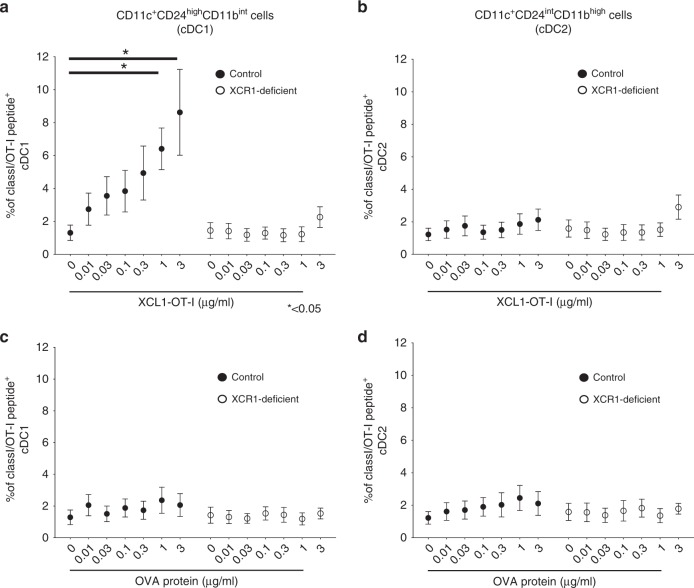
Selective targeting of XCL1-OT-I to XCR1^+^ DCs. **a**–**d** Flt3L-induced BM DCs from control (*Xcr1*^*+/venus*^, filled circles) or XCR1-deficient (*Xcr1*^*venus/venus*^, open circles) mice were incubated with indicated concentrations of XCL1-OT-I (**a, b**) or OVA protein (**c, d**) for 6 h, and analysed by a flow cytometer. Percentages of CD11c^+^CD24^high^CD11b^int^ cells (cDC1) (**a, c**) and CD11c^+^CD24^int^CD11b^high^ cells (cDC2) (**b, d**) expressing OT-I/H-2K^b^ complex are shown. Similar data were obtained from two independent experiments. *p < 0.05.

We further examined whether XCL1-OT-I leads to OT-I Ag presentation on XCR1^+^DCs. When stimulated with XCL1-OT-I at 1 µg/ml or more, OT-I/H-2K^b^-positive cells increased to more than 4% of CD11c^+^CD24^high^CD11b^int^ cells, which correspond to cDC1s, i.e. XCR1^+^ DCs from control mice (Fig. 2a). The increase was severely impaired in CD11c^+^CD24^high^CD11b^int^ cells from XCR1-deficient mice (Fig. 2a). Meanwhile, far fewer OT-I/H-2K^b^-positive cells were found in CD11c^+^CD24^int^CD11b^high^ cells, which correspond to cDC2s, i.e. XCR1^−^ DCs from control mice (Fig. 2b). When stimulated with OVA protein, OT-I/H-2K^b^-positive cells were not increased in either CD11c^+^CD24^high^CD11b^int^ or CD11c^+^CD24^int^CD11b^high^ cells (Fig. 2c, d). Thus, these results suggest that XCL1-OT-I leads to OT-I Ag presentation on cDC1s in an XCR1-dependent manner.

### XCL1-OT-I can induce potent CD8^+^ T-cell responses

**Fig. 3:**
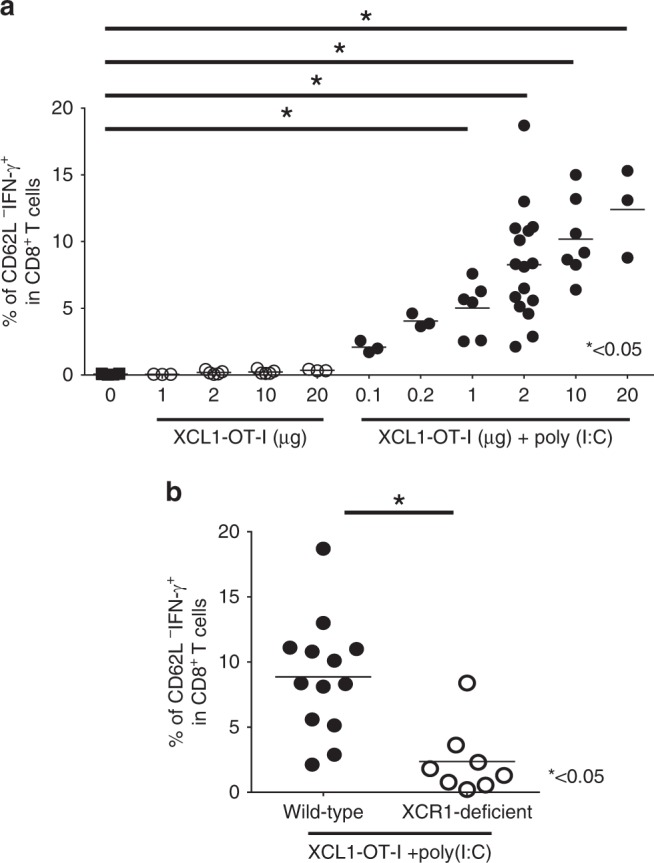
XCR1-dependent Ag-specific CD8^+^ T-cell responses induced by XCL1-OT-I. **a** C57B/6 J mice were immunised with the indicated concentrations of XCL1-OT-I with or without 20 µg of poly(I:C). **b** Wild-type or XCR1-deficient (*Xcr1*^*venus/venus*^, open circles) mice were immunised with 2 µg of XCL1-OT-I plus 20 µg of poly(I:C). Seven days after immunisation, splenocytes were stimulated with OT-I peptide and analysed by a flow cytometer. Percentages of IFN-γ-producing CD62L^−^ cells in CD8^+^ T cells are shown. The results of three independent experiments were combined. *p < 0.05, with Mann–Whitney U test.

To examine whether XCL1-OT-I can induce CD8^+^ T-cell responses, we analysed IFN-γ production from Ag-specific CD8^+^ T cells (Fig. 3a). Immunisation with up to 20 µg per mouse of XCL1-OT-I did not induce IFN-γ production from Ag-specific CD8^+^ T cells (Fig. 3a). We then chose a synthetic double-stranded RNA, poly(I:C), as an immune adjuvant that functions through Toll-like receptor 3 (TLR3) or cytosolic sensors, such as RIG-I.^34^ XCR1^+^ DCs respond to poly(I:C) mainly through TLR3.^35^ When immunised with poly(I:C), 0.2 µg or high doses of XCL1-OT-I could induce significant levels of Ag-specific CD8^+^ T-cell responses (Fig. 3a). To further examine if XCL1-OT-I-induced effects were dependent on XCR1, XCL1-OT-I plus poly(I:C) were injected to control or XCR1-deficient mice, and IFN-γ production from Ag-specific CD8^+^ T cells was measured. IFN-γ-producing cells were severely diminished in XCR1-deficient mice, indicating that XCL1-OT-I induced CD8^+^ T-cell responses through XCR1 (Fig. 3b).

**Fig. 4:**
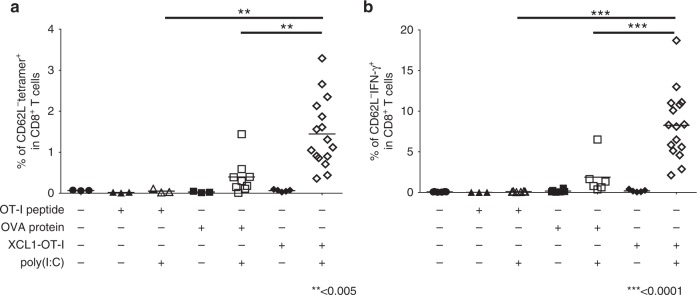
CD8^+^ T-cell responses induced by the OT-I peptide, OVA protein or XCL1-OT-I. **a**, **b** C57BL/6 J mice were immunised with 15 µg of OT-I peptide, 6.7 µg of OVA protein or 2 µg of XCL1-OT-I with or without 20 µg of poly(I:C). Seven days after immunisation, splenocytes were analysed by a flow cytometer. Ag-specific CD8^+^ T-cell responses were monitored by OT-I/H-2K^b^ tetramer staining (**a**), and intracellular staining of IFN-γ after in vitro stimulation with the OT-I peptide (**b**). The results were combined from three independent experiments. **p < 0.005. ***p < 0.0001, with ANOVA test.

Next, we compared CD8^+^ T-cell responses induced by XCL1-OT-I with those induced by the OT-I peptide or OVA protein. First, we have analysed the frequency of Ag-specific CD8^+^ T cells. Injection of 15 µg (16 µmol) per mouse of the OT-I peptide plus poly(I:C) did not induce any increase in Ag-specific CD8^+^ T cells. Injection of 6.7 µg (0.16 µmol) per mouse of OVA protein plus poly(I:C) could significantly induce an increase in Ag-specific CD8^+^ T cells, but the increase was lower than that induced by 2 µg (0.16 µmol) of XCL1-OT-I plus poly(I:C) (Fig. 4a). We have also analysed IFN-γ production from Ag-specific CD8^+^ T cells (Fig. 4b). Although IFN-γ production was observed when injected by OVA protein plus poly(I:C) or by XCL1-OT-I plus poly(I:C), IFN-γ- producing cells were in greater number upon injection of XCL1-OT-I plus poly(I:C) than upon injection of OVA protein plus poly(I:C). Thus, XCL1-OT-I could induce more potent CD8^+^ T-cell responses than OT-I or OVA protein.

### XCL1-OT-I can induce potent antitumour effects that depend on CD8^+^ T cells and XCR1

**Fig. 5:**
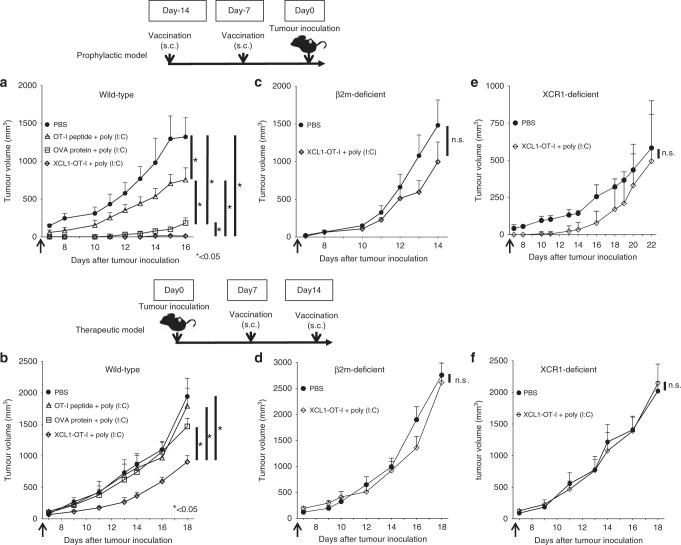
Antitumour effects of XCL1-OT-I. **a** C57BL/6J mice were unimmunised (PBS) or immunised with OT-I peptide, OVA protein or XCL1-OT-I plus poly(I:C) on 14 and 7 days before inoculation of B16-OVA. **b** C57BL/6J mice were inoculated with B16-OVA and unimmunised (PBS) or immunised with OT-I peptide, OVA protein or XCL1-OT-I plus poly(I:C) on 7 and 14 days after the inoculation of B16-OVA. **c** β2m-deficient mice were unimmunised (PBS) or immunised with XCL1-OT-I plus poly(I:C) on 14 and 7 days before inoculation of B16-OVA. **d** β2m-deficient mice were inoculated with B16-OVA and unimmunised (PBS) or XCL1-OT-I plus poly(I:C) on 7 and 14 days after the inoculation of B16-OVA. **e** XCR1-deficient *(Xcr1*^*venus/venus*^) mice were unimmunised (PBS) or immunised with XCL1-OT-I plus poly(I:C) on 14 and 7 days before inoculation of B16-OVA. **f** XCR1-deficient *(Xcr1*^*venus/venus*^) mice were inoculated with B16-OVA, and unimmunised (PBS) or immunised with XCL1-OT-I plus poly(I:C) on 7 and 14 days after inoculation of B16-OVA. **a**, **c**, **e** The prophylactic model. **b**, **d**, **f** The therapeutic model. Tumour volumes were calculated at indicated days (n = 5 for each group). Similar data were obtained from one or two independent experiments. *p < 0.05, with ANOVA test. n.s., not significant. s.c., subcutaneously.

We analysed the antitumour effects of OT-I peptide, OVA protein and XCL1-OT-I plus poly(I:C) in the prophylactic model. Without immunisation, tumours appeared as a macroscopic mass around 9 days after the tumour inoculation. Immunisation of both OT-I peptide plus poly(I:C) and OVA protein plus poly(I:C) inhibited tumour growth, although the latter showed more prominent inhibitory effects than the former. Notably, when immunised with XCL1-OT-I plus poly(I:C), tumour mass was hardly detected until 12 days, and the tumour growth was much more prominently inhibited throughout the observed periods than the other immunisations (Fig. 5a).

We next analysed whether XCL1-OT-I can suppress the tumour growth in the therapeutic model. On 7 and 14 days after the tumour inoculation, mice were immunised with the OT-I peptide, OVA protein or XCL1-OT-I plus poly(I:C). Tumour growth was not affected by injection of either OT-I peptide plus poly(I:C) or OVA protein plus poly(I:C). In contrast, when injected with XCL1-OT-I plus poly(I:C), tumour growth was significantly inhibited at day 18 (Fig. 5b). Taken together, these results show that XCL1-OT-I is effective as a cancer vaccination, and is more superior to immunisation with the Ag peptide or protein in both prophylactic and therapeutic models.

We then analysed whether the antitumour effects of XCL1-OT-I depend on CD8^+^ T cells. In β2m-deficient mice, CD8^+^ T cells were absent due to abolished MHC class I expression.^36^ XCL1-OT-I plus poly(I:C) did not inhibit the tumour growth in β2m-deficient mice in either prophylactic or therapeutic models (Fig. 5c, d). The results suggest that XCL1-OT-I-induced antitumour effects were dependent on CD8^+^ T cells.

We also analysed whether XCR1 is involved in the antitumour effects of XCL1-OT-I. In XCR1-deficient mice, tumour growth inhibition by XCL1-OT-I plus poly(I:C) was abolished in both prophylactic and therapeutic models (Fig. 5e, f). The results suggest that XCL1-OT-I showed its antitumour effects through XCR1.

### XCL1-OT-I can improve the antitumour effects of the ICIs

**Fig. 6:**
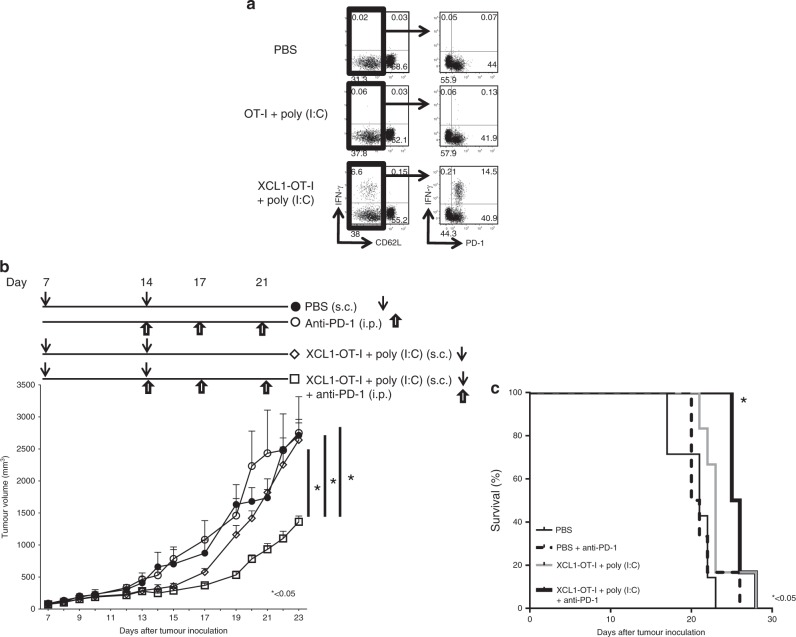
Effects of XCL1-OT-I in combination with anti-PD-1 Ab. **a** Expression of an immune checkpoint molecule, PD-1, on CD8^+^CD62L^−^ T cells. Mice were unimmunised (PBS) or immunised with OT-I peptide or XCL1-OT-I plus poly(I:C). Seven days after immunisation, splenocytes were prepared, stimulated with OT-I and analysed by a flow cytometer. Representative dot plots are shown. **b**, **c** Effects of XCL1-OT-I and anti-PD-1 Ab in the therapeutic tumour model. Mice were inoculated with B16-OVA on day 0, and immunised with PBS only, or 2 µg of XCL1-OT-I plus 20 µg of poly(I:C) on days 7 and 14. Anti-PD-1 Ab was administered on days 14, 17 and 21. Tumour volumes at indicated days and survival analysis are shown (n = 5 for each group). Similar data were obtained from two independent experiments. *, **p < 0.05, with ANOVA test. s.c., subcutaneously. i.p., intraperitoneal.

We then examined whether immunisation with XCL1-OT-I can enhance the antitumour effects of ICIs. We first examined expression of PD-1, an immune checkpoint molecule, on CD8^+^ T cells upon immunisation with the OT-I peptide or XCL1-OT-I plus poly(I:C) (Fig. 6a). IFN-γ-producing cells were hardly detected in unimmunised mice or mice immunised with OT-I peptide plus poly(I:C). However, IFN-γ-producing cells increased after immunisation with XCL1-OT-I plus poly(I:C), and PD-1 was expressed on more than 90% (this was calculated by dividing 14.5% by 14.5 plus 0.21% in the right lowermost dot plot of Fig. 6a of the activated IFN-γ-producing CD8^+^ T cells). Thus, PD-1 expression was augmented in activated IFN-γ-producing CD8^+^ T cells upon immunisation with XCL1-OT-I plus poly(I:C).

This enhanced expression of PD-1 suggests that XCL1-OT-I can augment the antitumour effects of ICIs. To prove this, we immunised the tumour-bearing mice with XCL1-OT-I twice with a 1-week interval, and evaluated the effects of anti-PD-1 Ab (Fig. 6b). Injection of XCL1-OT-I plus poly(I:C) on days 7 and 14 inhibited the tumour growth at day 15, but the inhibitory effects were not prominent on day 17 or later. Meanwhile, injection of XCL1-OT-I plus poly(I:C) followed by subsequent injection of anti-PD-1 Ab significantly decreased the tumour size at day 23 after tumour inoculation (*p* < 0.05) and prolonged survival (*p* < 0.05) than injection of XCL1-OT-I plus poly(I:C) without anti-PD-1 Ab (Fig. 6c). Thus, XCL1-OT-I enhanced the antitumour effects with an ICI, anti-PD-1 Ab.

## Discussion

We aimed to deliver a cancer Ag peptide to XCR1^+^ DCs with high cross-presenting activity, by using a fusion protein, XCL1-OT-I, to carry the OT-I peptide and a chemokine, XCL1. Injected with an immune adjuvant, poly(I:C), XCL1-OT-I elicited potent CD8^+^ T-cell responses, and showed more prominent antitumour effects than the OT-I peptide or OVA protein in both, prophylactic and therapeutic tumour models. Furthermore, pre-immunisation with XCL1-OT-I plus poly(I:C) enhanced the antitumour effects of an ICI, anti-PD-1.

When injected with poly(I:C), XCL1-OT-I induced significant CD8^+^ T-cell responses at 0.2 µg or higher doses per mouse (Fig. 3a). As a sufficient dose for significant effects, throughout the experiments, XCL1-OT-I was used at 2 µg per mouse. For comparison, OVA protein was used at the same moles as XCL1-OT-I, while OT-I peptide was used at 100 times more moles of XCL1-OT-I. By using these amounts for immunisation, XCL1-OT-I consistently shows higher CD8^+^ T-cell responses and antitumour effects than OVA protein or OT-I peptide (Figs. 4 and 5). Thus, in terms of the efficiency per mole, XCL1-OT-I is more effective than the OT-I peptide or OVA protein.

Potent antitumour activity of XCL1-OT-I should come from its ability to target Ag to XCR1^+^ DCs, which are crucial Ag-presenting cells for generation of CD8^+^ T-cell responses against cancers. XCL1-OT-I retained the chemotactic activity of XCL1, though the OT-I peptide is attached to XCL1 (Fig. 1d), ensuring that it can be targeted to XCR1. After XCL1-OT-I is targeted to XCR1^+^ DCs, it is presumed to be incorporated and processed in XCR1^+^ DCs, and presented as MHC class I-restricted OT-I Ag on XCR1^+^ DCs. This is supported by the finding that OT-I Ag is presented by cDC1, but not by cDC2, and that this OT-I Ag presentation was abolished in the absence of XCR1 (Fig. 2a, b). Compared with XCL1-OT-I, OVA protein induced much lower presentation of OT-I Ag on cDC1s (Fig. 2c), indicating that OT-I Ag presentation induced by XCL1-OT-I depends on XCR1, and that XCR1 can incorporate its ligand and process it for Ag presentation. This XCR1-dependent presentation should lead to antitumour effects of XCL1-OT-I, because not only CD8^+^ T-cell responses but also antitumour effects induced by XCL1-OT-I were severely decreased in XCR1-deficient mice (Figs. 3b, 5e, f). Thus, like monoclonal antibodies against an endocytic receptor such as DEC205,^17,18^ XCL1-based targeting through XCR1 can mediate Ag processing and presentation, thereby leading to CD8^+^ T-cell responses against tumours.

XCL1-OT-I plus poly(I:C) induced PD-1 expression on more than 90% of activated IFN-γ-producing CD8^+^ T cells (Fig. 6a). This augmented expression of PD-1 might limit the antitumour effects of XCL1-OT-I, but this also suggests that pretreatment with XCL1-OT-I can be useful as a combination therapy with ICIs. As expected, pretreatment with XCL1-OT-I plus poly(I:C) could significantly enhance the antitumour effects of ICIs (Fig. 6b).

Previous reports showed that fusion vaccines composed of Ag proteins with XCL1 can be targeted and loaded to cDC1s or XCR1^+^ DCs, and elicit Ag-specific CD8^+^ T-cell responses.^23–25^ Fossum et al. have shown that the intramuscular injection of DNA vaccine encoding the Ag protein fused with XCL1 can provoke Ag-specific CD8^+^ T-cell responses and induce prophylactic effects against influenza virus infection.^23^ This DNA vaccination has not been tested in tumour models, although theoretically it should be effective. Two studies addressed the effects of the fusion protein in tumour models. Hartung et al. immunised mice by intravenous injection of the fusion protein composed of an Ag protein with XCL1, and observed its prophylactic effects against cancers.^24^ Terhorst et al. performed laser-assisted intradermal delivery of the fusion protein composed of an Ag protein with XCL1, and showed its prophylactic and therapeutic effects against cancers.^25^ In this study, we generated a fusion protein of an Ag peptide instead of an Ag protein, and showed that subcutaneous injection of the fusion protein can induce anticancer effects in both prophylactic and therapeutic tumour models (Fig. 5a, b). Compared with most Ag proteins fused with XCL1, an Ag peptide fused with XCL1 should be small enough to be synthesised chemically, and can be more readily applied to the human system, considering that human XCL1 protein consists of 92 amino acids. Furthermore, at clinical situations, subcutaneous injection is widely used, and should be preferable not only to intravenous injection but also to laser-assisted intradermal delivery. We have further shown that the fusion protein of an Ag peptide and XCL1 enhanced the anticancer effects of ICIs in the therapeutic tumour model.

Currently several cancer vaccines depend on DC activation. Sipuleucel-T is an active cellular immunotherapy aimed at the treatment of prostate cancers, and involves autologous peripheral-blood mononuclear cells, including Ag-presenting cells, activated ex vivo by a fusion protein consisting of a cancer Ag, prostatic acid phosphatase^37^ and a DC-activating granulocyte–macrophage colony-stimulating factor (GM-CSF). GVAX is an allogeneic whole-cell vaccine, consisting of cell lines producing GM-CSF. However, no vaccines are available targeting specifically to a DC subset.^38^ Human XCR1 is expressed in one DC subset, CD141^+^/BDCA3^+^ DC, which is known for possessing high cross-presenting activity.^39,40^ XCL1 and its close relative, XCL2, are ligands for human XCR1, so Ags fused with XCL1 or XCL2 should be targeted to CD141^+^/BDCA3^+^ DC to provoke Ag-specific CD8^+^ T-cell responses in human. Targeting XCR1^+^ DCs by a fusion protein of any type of Ags, including neoantigens and human XCL1/2, should have the potential to be a promising anticancer vaccine in combination with ICIs.

## Data Availability

The raw datasets analysed for this study are available from the corresponding authors on reasonable request.
